# Efficient Separation of Proteins and Polysaccharides from *Dendrobium huoshanense* Using Aqueous Two-Phase System with Ionic Liquids

**DOI:** 10.3390/molecules27165284

**Published:** 2022-08-18

**Authors:** Peipei Yang, Mengya Lu, Jing Zhao, Emelda Rosseleena Rohani, Rongchun Han, Nianjun Yu

**Affiliations:** 1 School of Pharmacy, Anhui University of Chinese Medicine, Hefei 230012, China; 2 Laboratory of Quality Assessment, Shenyang Institute of Food and Drug Control, Shenyang 110122, China; 3 Institute of Systems Biology, University Kebangsaan Malaysia, Bangi 43600, Malaysia

**Keywords:** *Dendrobium huoshanense*, ionic liquid, polysaccharides, aqueous two-phase system

## Abstract

By applying the hydrophilic ionic liquid, 1-butyl-3-methylimidazolium chloride ([C_4_mim]Cl), and inorganic salts (K_3_PO_4_), an ionic liquid aqueous two-phase system (ILATPS) was established for the separation of *Dendrobium huoshanense* polysaccharides (DhPs) and proteins. The effects of inorganic salt concentration, IL quantity, crude DhPs concentration, pH value and temperature were studied to achieve the optimal condition. With the best combination of ILATPS (1.75 g K_3_PO_4_, 1.25 g [C_4_mim]Cl, 10 mg crude DhPs and 5.0 mL ddH_2_O at pH 7.0 under 25 °C), the extraction efficiency rates for DhPs and proteins were 93.4% and 90.2%, respectively. The processed DhPs retrieved from the lower salt-rich phase comprised mannose, glucose, galactose, arabinose, and galacturonic acid with a molar ratio of 185:71:1.5:1:1 and the molecular weight was 2.14 × 10^5^ Da. This approach is fast, simple and environmentally friendly. It provides a new insight into purifying functional polysaccharides of plant origin.

## 1. Introduction

As a type of macromolecular polymer, polysaccharides are present in all living cells [1]. With the typical composition of more than 10 monosaccharides bounded together by glycosidic linkages, the structure can be linear or highly branched and the molecular weight ranges from tens of thousands to a million Dalton and more [2]. According to the species of monosaccharide as building blocks, polysaccharides can be divided into two forms: homopolysaccharides and heteropolysaccharides [3]. Regarding plants, the function of polysaccharides is mainly related to structure, e.g., cellulose, or storage, e.g., starch. However, they also play an important role in other biological processes such as cell communication, molecular recognition, cell adhesion, responding to cold stress, drought stress and so forth [4,5,6,7]. In terms of therapeutic applications, plant polysaccharides have been tested and proven throughout history to have anti-inflammatory, anti-oxidation, immunomodulation, anticancer and anti-diabetic effects [8,9,10]. For example, oxygen as the indispensable ingredient for most living organisms on Earth is highly reactive and can damage cellular components including DNA and proteins. Finding antioxidant metabolites such as plant polysaccharides to counteract excessive oxidation is meaningful in preventing and treating certain diseases. As a result, pachman, fucoidan, polysaccharides from *Panax ginseng*, *Coriolus versicolor* as well as *Astragalus memeranaceus* are now drugs sold in drugstores. In contrast, with ample evidence of polysaccharides’ efficacy, the number of commercial products is limited [11], partially due to difficulties in separation and purification.

Among the various approaches applied to extract, separate and purify plant crude polysaccharides, extraction using water and ethanol remains the most common and classic method and is adopted widely in scientific research laboratories and industry. This method involves cleaning, homogenization, separation and centrifugation steps [12]. After the powdered raw material is extracted in hot water, the supernatant containing polysaccharides is aspirated to mix with absolute ethanol at a certain ratio to yield precipitate that will then be centrifuged and collected for purification. In practice, gradient precipitation by ethanol is frequently used. Nevertheless, the contents of precipitated protein also increase with raised ethanol volume [13]. To decipher the structure and to assess the function of polysaccharides, proteins have to be removed before downstream operations such as ion exchange, dialysis, gel filtration and affinity chromatography, etc. Conventionally, deproteinization requires organic solvents that are usually hazardous with the operation laborious. Taking Sevag reagent, for example, chloroform-n-butanol can be added to the crude extract and after shaking vigorously, colloidal denatured protein will form between the water and organic solvent layer for subsequent removal. However, such an approach often suffers a major drawback of repetitive operation and low efficiency as well [14].

Compared with conventional separation operations, the aqueous two-phase system (ATPS) as a liquid–liquid fractionation method has attracted considerable attention recently because its characteristics include its low cost, ease to scale up, capability of continuous operation and being environmentally friendly [15,16]. Since its discovery in 1896 by the Dutch botanist Martinus Beijerinck who accidentally mixed the aqueous solution of gelatin and starch, ATPS has evolved from mixing a wide range of chemicals in water to two-polymer or polymer-salt systems, which are being intensively studied for efficient purification of biomolecules [17]. Concerning ATPS, water is the main constituent in both phases to provide a gentle environment for the separation of distinct molecules with the polymers stabilizing their structure [18]. To ensure effective separation, suitable polymers must be considered carefully for most phase-forming polymers with high viscosity create a nontransparent solution obstructing the determination of target compounds. As an alternative approach, Rogers et al., established a novel ATPS adopting hydrophilic ionic liquid (IL) in the presence of certain inorganic salts [19]. The new system excels in the following aspects: high extraction efficiency, usage of innocuous solvent, low viscosity, swift phase separation and less emulsion formation [14,20]. Several experiments were successfully undertaken by following the principle of this method to separate antibiotics, proteins and alkaloids [21,22]. Furthermore, this system was also proven to be efficient in separating carbohydrates and proteins with the upper IL-rich layer containing most of the proteins and the lower salt-rich phase holding the majority of the polysaccharides [23].

In this study, water solution containing inorganic salts K_3_PO_4_ was added to 1-butyl-3-methylimidazolium chloride ([C_4_mim]Cl) to form an IL aqueous two-phase system (ILATPS) for the purpose of separating polysaccharides (DhPs) and proteins extracted from stems of *Dendrobium huoshanense*, a folk medicine originated from China with polysaccharide as its main active compound. The extraction efficiency was first tested using bovine serum albumin (BSA) and parameters affecting the final yield of polysaccharides were interrogated in order to pinpoint the optimal separation conditions. The composition and preliminary structure of the purified polysaccharides were also analyzed. The results demonstrated that ILATPS featuring [C_4_mim]Cl as well as K_3_PO_4_ under related optimal conditions successfully separates *D. huoshanense* polysaccharides from proteins.

## 2. Results and Discussion

### 2.1. Optimal Conditions for ILATPS

#### 2.1.1. Determination of the Suitable Salt Type and Its Concentration Using BSA Solution

The appropriate type of inorganic salt at a suitable concentration is indispensable for ILATPS, which does not automatically form two layers. The addition of NaCl, KCl, K_2_SO_4_, Na_2_SO_4_ or KH_2_PO_4_ failed to cause phase separation. Due to stronger acting forces with water molecules, anions including PO_4_^3−^, HPO_4_^2−^, CO_3_^2−^ and OH^−^ help to form two phases in ILATPS. Meanwhile, ionic Gibbs free energy of hydration also plays an important role in forming layers concerning the interactions between inorganic salts and [C_4_mim]Cl. Our study tested relevant salts and demonstrated their phase-forming ability followed such order: K_3_PO_4_ > K_2_HPO_4_ > Na_2_CO_3_ > KOH. This was in line with a previous report that mainly focused on phosphate-based salts whose ability to induce the formation of liquid –liquid biphasic systems in presence of IL was considerable [21].

**Figure 1:**
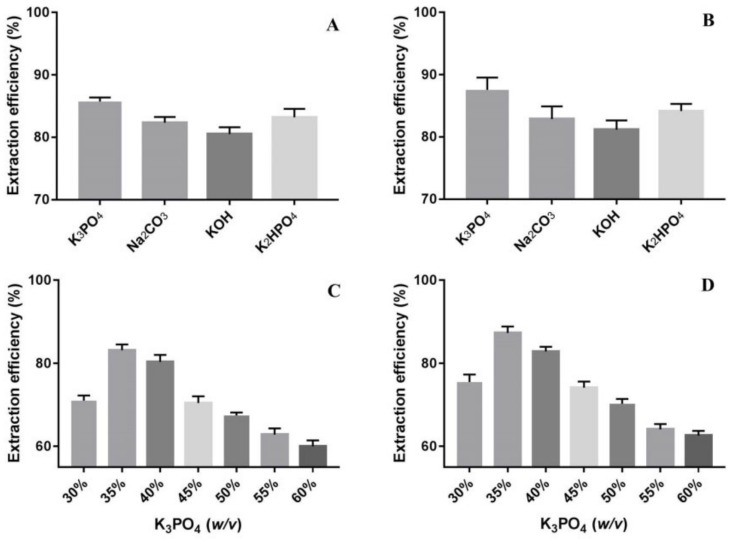
Comparison of protein extraction efficiency using different inorganic salts (**A**), different inorganic salts with pH values adjusted to 9.0 (**B**), different concentrations of K_3_PO_4_ with the following experimental condition: 1.0 g IL, 10 mg BSA and 5.0 mL ddH_2_O under room temperature with pH values not controlled (**C**), and different concentrations of K_3_PO_4_ with pH values adjusted to 9.0 (**D**). All data in Figure 1 are presented as mean with SD and *n* = 3 per group.

BSA was used in various IL systems including Ammoeng^TM^ 110, [C_6_mim]Br and [C_8_mim]Br to evaluate the protein partitioning capability of the ionic liquids [24]. To simplify our experiments in the search for suitable extraction parameters, we chose BSA as the starting material to test whether [C_4_mim]Cl was capable of extracting proteins from the lower salt-rich phase. By using 10 mg BSA, 5.0 mL ddH_2_O, 1.0 g [C_4_mim]Cl and 1.75 g inorganic salts under room temperature, protein extraction efficiency was as follows: K_3_PO_4_ > K_2_HPO_4_ > Na_2_CO_3_ > KOH (Figure 1A). All the four tested salts helped the ILATPS to extract more than 80% of BSA, with K_3_PO_4_ showing an extraction efficiency of 85.7% (Figure 1A). Because a fixed amount of inorganic salts will cause distinct pH values in the aqueous system, in the subsequent test, pH values for the four salts were adjusted to 9.0 and it was found that K_3_PO_4_ performed better (87.6%) in contrast with the other three salts (Figure 1B). Compared with less-charged anion species under suitable pH values (e.g., pH > 12.3), the hydrogen bond formed between K_3_PO_4_ and water was indeed stronger. Therefore, K_3_PO_4_ was chosen as the salting out reagent for the following studies.

To evaluate the influence of salt quantity on extraction efficiency, different concentrations of K_3_PO_4_ from 30% to 60% (*w*/*v*) were analyzed. Concentrations less than 30% (*w*/*v*) resulted in poor phase separation. For the tested range, when pH values were not controlled, the highest extraction capacity for BSA was observed at 35% (*w*/*v*) with an extraction efficiency of 83.1% (Figure 1C). When the pH values of ILATPS with different concentrations of K_3_PO_4_ were adjusted to 9.0, protein extraction efficiency was generally better (Figure 1D) compared with uncontrolled groups, suggesting lower pH values favor protein extraction in ILATPS containing K_3_PO_4_. From Figure 1C,D, it was found that K_3_PO_4_ at a concentration of 35% (*w*/*v*) performed best in extracting the tested proteins. Furthermore, with increased K_3_PO_4_ concentration, the solubility of carbohydrates would decrease due to competition among K^+^, PO_4_^3−^ and DhPs for water molecules by intermolecular hydrogen bonds. The intensifying competition caused fewer polysaccharides allocated into the lower salt-rich phase and therefore jeopardized the extraction efficiency from the aspect of carbohydrate [25]. Based on the findings above, ILATPS containing 35% (*w*/*v*) K_3_PO_4_ was determined.

#### 2.1.2. Determination of Optimal IL Quantity Using Crude DhPs Solution

**Figure 2:**
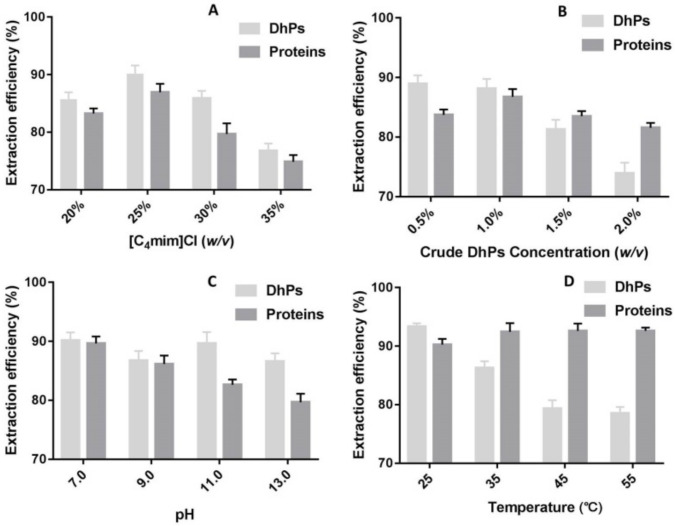
Determination of optimal quantity of [C_4_mim]Cl (**A**), crude DhPs concentration (**B**), pH value (**C**) and temperature (**D**) using the ILATPS system for extraction of polysaccharides and proteins from crude DhPs. All data in Figure 2 are presented as mean with SD and *n* = 3 per group.

By searching Sigma-Aldrich’s official website using the keyword “ionic liquid”, 350 products were retrieved. More and more ionic liquids are also being synthesized for their versatile applications. In this study, we chose [C_4_mim]Cl as the subject for its relatively low price and easy availability. Figure 2A showed the influence of IL quantity in ILATPS on DhPs and protein extraction efficiency. For both DhPs and proteins, similar trends were observed regarding extraction efficiency, which rose with an increased amount of [C_4_mim]Cl in ILATPS and then dropped with even more IL added to the system. The highest extraction efficiency for DhPs and proteins was 90.2% and 86.9%, respectively, when the IL proportion was 25% (*v*/*w*) in the system. Imidazolium cation of [C_4_mim]Cl features the aromatic π system and the π−π interaction between the aromatic residues of the proteins and the imidazolium cation may well be the driving force for the successful separation of proteins [26]. With the increased amount of IL in the upper layer, free space available to allocate the proteins in that phase will decrease. As a rule of thumb, [C_4_mim]Cl traps up to 15% of water. That is to say, every 1.0 g [C_4_mim]Cl absorbs 0.15 mL ddH_2_O. The observed decreasing extraction efficiency for DhPs at concentrations of 30% and 35% could be because the water in the upper IL-rich layer contains a small number of DhPs and interferes with protein extraction. Follow-up studies are needed in probing the in-depth mechanisms of ILATPS. Taken together, 25% (*w*/*v*) IL was used to resolve other optimal parameters.

#### 2.1.3. Determination of Suitable Crude DhPs Concentration

Hydroxyl groups of DhPs interact with and weaken the hydrogen bonds that naturally hold different water molecules. A newly emerged hydrogen bond system between hydroxyl groups of DhPs and H_2_O will in turn decrease the number of water molecules capable of interacting with, and dissolving, protein molecules that are forced to enter the upper layer consisting of [C_4_mim]Cl. The inter-molecular hydrogen bonding between DhPs and H_2_O keeps polysaccharides in the lower phase. DhPs concentrations at both 0.5% (*w*/*v*) and 1.0% (*w*/*v*) achieved high carbohydrate extraction efficiency of 89.0% and 88.2%, respectively (Figure 2B). Nevertheless, concentrations at 1.5% (*w*/*v*) and 2.0% (*w*/*v*) resulted in decreased extraction efficiency for DhPs. A suitable amount of DhPs forms hydrogen bonds with H_2_O adequately, while excessive carbohydrates cause the aggregation of DhP molecules and the growing intra-molecular hydrogen bonds sabotage the interactions between DhPs and water molecules. Concerning extraction efficiency for proteins, different concentrations of crude DhPs showed less impact compared with that of polysaccharides; although, slight declination was observed with the addition of extra DhPs. With similar extraction efficiency, a higher concentration of initial crude polysaccharides was preferred. The suitable DhPs concentration in ILATPS was set to 1.0% (10 mg/mL).

#### 2.1.4. Determination of Optimal pH

For the separation system containing K_3_PO_4_, ILATPS does not form two layers with pH below 7.0 or above 13.0, and therefore pH values at 7.0, 9.0, 11.0 and 13.0 were prepared for investigation. The reagent for adjustment of pH was H_3_PO_4_ to avoid the introduction of unrelated ions. The extraction efficiency for DhPs was stable and above 86.0% at all designated conditions. However, extraction efficiency decreased rapidly for proteins with increased pH values (Figure 2C). Isoelectric points of *D. huoshanense* proteins and the pH of the solution determine the charged state of the proteins. Electrostatic interactions between the ionic groups of [C_4_mim]Cl and the charged groups of protein have a vital influence in determining the optimal pH of the aqueous solutions for the best extraction efficiency. The main purpose of this study was to find an efficient approach to removing proteins that came along with the bioactive *D. huoshanense* polysaccharides. Therefore, optimal pH was controlled at 7.0.

#### 2.1.5. Determination of Optimal Temperature

The temperature could be a significant factor regarding extraction efficiency for various biomolecules adopting ILATPS [26,27]. Different temperatures (25, 35, 45 and 55 °C) were tested to evaluate their impact on the separation efficiency of DhPs and proteins. For the extraction of DhPs, the highest efficiency was found at 25 °C, indicating room temperature facilitated separation of DhPs in ILATPS using [C_4_mim]Cl compared with higher temperatures. This was also in line with the previous report by Tan et al. [28]. As far as protein extraction was concerned, contents of protein remained in the lower salt-rich phase and slightly decreased at higher temperatures with extraction efficiency increasing from 90.2% at 25 °C to 92.6% at 55 °C (Figure 2D). Despite the trend, the extraction efficiency for *D. huoshanense* protein under various temperatures showed no statistical significance (*p* = 0.089). Judging from the result, the process for extracting proteins is endothermic and higher temperatures raise protein extraction efficiency to a limited extent. However, DhPs were more stable under room temperature, and high temperatures significantly sabotage DhPs extraction (Figure 2D). Hence, partitioning procedures were undertaken at 25 °C.

### 2.2. DPPH Scavenging Capacity of Crude and Processed DhPs

**Figure 3:**
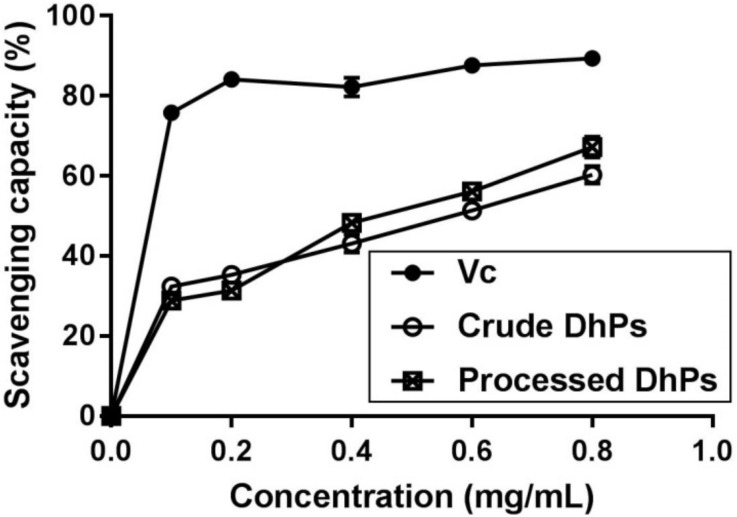
Determination of DPPH free radical scavenging capacity. All data in Figure 3 are presented as mean with SD and *n* = 3 per group.

DPPH (2,2-Diphenyl-1-picrylhydrazyl) free radicals have strong absorption at 517 nm and based on this, DPPH scavenging capacity tests on both crude and processed DhPs were established [29]. With the presence of free radical scavengers, single-electron pairing causes decreased absorption at 517 nm, and the fading degree is correlated with the number of electrons scavengers provide [30]. Therefore, quantitative analysis can be performed using a spectrophotometer. The scavenging capacity of crude, processed DhPs, as well as vitamin C (Vc) on DPPH free radicals, is demonstrated in Figure 3. As the positive control, the clearance effect of Vc was remarkable, starting with 75.8% at the concentration of 0.1 mg/mL and achieving 89.4% at 0.8 mg/mL. It was found that after purification, polysaccharides from *D. huoshanense* retained their bioactivity as far as DPPH scavenging capacity was concerned. Both crude and processed DhPs displayed a scavenging effect; although, their capacity was not as strong as that of Vc. At low concentrations, the scavenging capability of crude DhPs was higher than processed DhPs. At the concentration of 0.8 mg/mL, the scavenging rates of crude and processed DhPs were 60.3% and 67.2%, respectively. The above results suggested that the DPPH free radical scavenging capacity of DhPs was enhanced in a certain concentration range (0.4, 0.6 and 0.8 mg/mL) after purification adopting ILATPS.

### 2.3. Preliminary Structural Determination for DhPs

**Figure 4:**
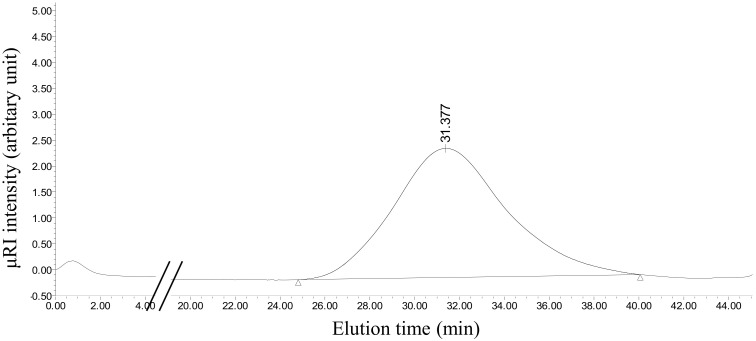
Elution pattern of the processed DhPs in 0.05 M NaCl solution at 40 °C using HPGPC.

**Figure 5:**
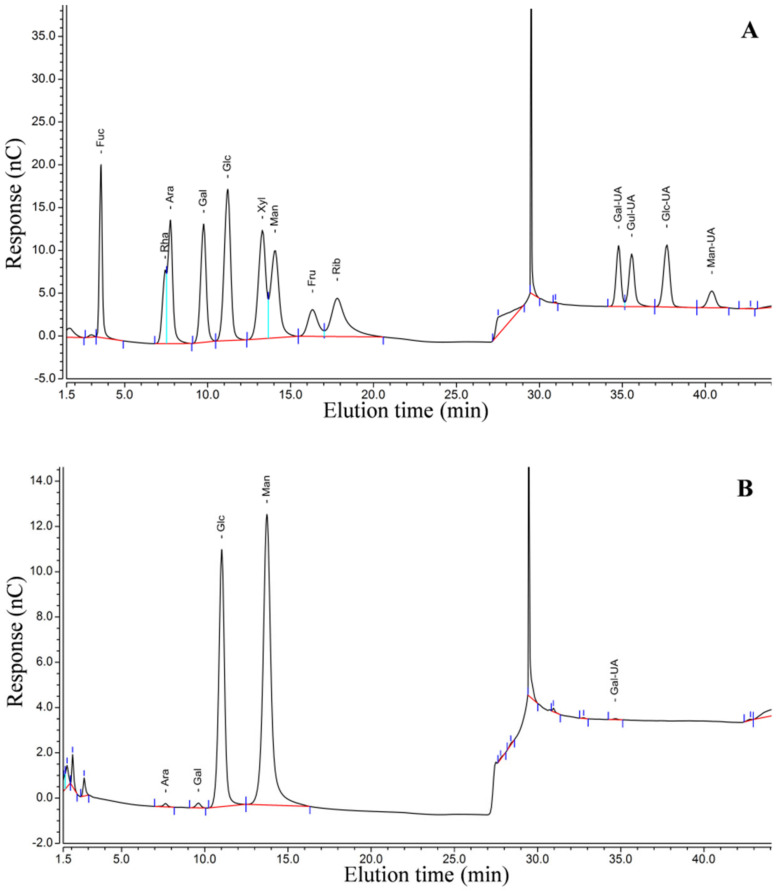
Ion chromatographic profile of monosaccharide standards (**A**) and the processed DhPs (**B**). Peaks: Ara (Rt: 7.76 min); Gal (Rt: 9.77 min); Glc (Rt: 11.22 min); Man (Rt: 14.08 min); Gal-UA (Rt: 34.87 min). Note that the chromatographic peak at ca 30 min represents sodium acetate (NaAc).

Under optimal conditions, DhPs extracted from the lower phase were partially purified with dialysis, precipitation by adding 5 volumes of absolute ethanol, centrifugation and freeze-drying. High-performance gel permeation chromatography (HPGPC) was adopted for the evaluation of homogeneity as well as the molecular weight of the processed DhPs. The HPGPC profile demonstrated a single and symmetrical peak, indicating the tested compound was homogeneous (Figure 4). The weight (Mw), the number-average molecular weight (Mn) and the molecular weight distribution (Mw/Mn) of the processed DhPs in 0.05 M NaCl solution were 2.14 × 10^5^ Da, 1.54 × 10^5^ Da and 1.38, respectively. Including fucose (Fuc), rhamnose (Rha), arabinose (Ara), galactose (Gal), glucose (Glc), xylose (Xyl), mannose (Man), fructose (Fru), ribose (Rib), galacturonic acid (Gal-UA), glucuronic acid (Glc-UA), mannuronic acid (Man-UA) and guluronic acid (Gul-UA), thirteen monosaccharide standards were used to establish the reference chromatography (Figure 5A). The purity of all standard substances was ≥97%. Monosaccharide composition analysis suggested that the processed DhPs were comprised of Man, Glc, Gal, Ara and Gal-UA with a molar ratio of 185:71:1.5:1:1 (Figure 5B). This is in line with the previous report in which types of monosaccharides in different parts of *D. huoshanense* varied, but mannose and glucose were the most common components in all studied organs [31].

**Figure 6:**
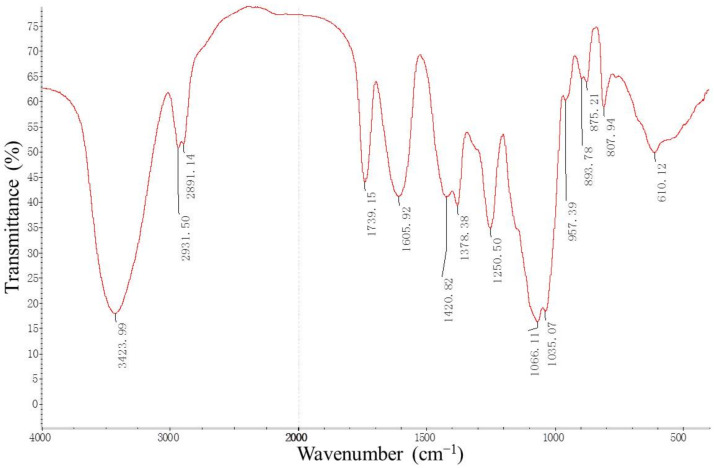
The FT-IR spectrum of the processed DhPs.

From the FT-IR spectrum of processed DhPs (Figure 6), three typical absorption peaks of polysaccharides were detected. The first was a wide and strong stretching peak at 3423.99 cm^−1^ indicating the O-H stretching vibration. The second and the third were the weak absorption peaks at 2931.50 cm^−1^ and 2891.14 cm^−1^, respectively, for the C-H (-CH_3_ and -CH_2_) stretching vibration. Peaks ranging from 1450 to 1200 cm^−1^ (1420.82 cm^−1^, 1378.38 cm^−1^ and 1250.50 cm^−1^) indicated C-H deviational vibration. Peaks at 1066.11 cm^−1^ and 1035.07 cm^−1^ proved again the existence of pyranoside. The peak at 893.78 cm^−1^ was ascribed to the β-type glycosidic linkages in the polysaccharides.

### 2.4. Recovery of [C_4_mim]Cl

[C_4_mim]Cl concentrated at the upper layer in ILATPS will cause pollution to the sewage system if it is left untreated. Because of its water solubility and low biodegradability, [C_4_mim]Cl is a potential long-lasting aquatic pollutant. At the same time, recycling the IL will reduce the cost substantially especially when such efforts are conducted on an industrial scale because the price for IL is still very high compared with old-fashioned extractants. The solution (20 mL) collected from the upper layer was dried in a water bath and the residue was dissolved in 80 mL dichloromethane (CH_2_Cl_2_) solution that was subsequently evaporated to obtain [C_4_mim]Cl with a recovery rate of 92.8%. In doing so, we have to admit that recovering [C_4_mim]Cl is at the cost of releasing CH_2_Cl_2_, which, in turn, has a significant environmental impact. Different volumes of CH_2_Cl_2_ were tested and it was found that when 45 mL dichloromethane was used to process 20 mL IL solution, a recovery rate of >90% could still be achieved.

## 3. Materials and Methods

### 3.1. Materials and Chemicals

Three-year-old stems of *D. huoshanense* were sampled from the greenhouse of Anhui University of Chinese Medicine. Stems were freeze-dried on an LGJ-10 lyophilizer (Songyuan, China) and then ground to fine powder for extraction of crude DhPs according to Chinese Pharmacopoeia [32]. [C_4_mim]Cl with ≥99% purity, DPPH (≥97% purity), vitamin C (≥99% purity), dextran polymers and monosaccharide standards (all ≥96% purity) including fucose (Fuc), rhamnose (Rha), arabinose (Ara), galactose (Gal), glucose (Glc), xylose (Xyl), mannose (Man), fructose (Fru), ribose (Rib), galacturonic acid (Gal-UA), glucuronic acid (Glc-UA), mannuronic acid (Man-UA) and guluronic acid (Gul-UA) were obtained from Sigma-Aldrich, St. Louis, MO, USA. BSA (≥99% purity); Coomassie Brilliant Blue G-250 (≥90% purity) and trifluoroacetic acid (TFA, ≥99% purity) were purchased from Sangon Biotech, Shanghai, China. All other solvents and chemicals, such as K_3_PO_4_, K_2_HPO_4_, NaOH and HCl, were of laboratory grade.

### 3.2. Extraction of Crude DhPs

Stem powder (50 g) of *D. huoshanense* was treated with refluxing petroleum ether for 4 h to eliminate pigments and lipids. After the residue was freeze-dried, the hot water extraction method was applied [33]. In brief, 1 g of dried *D. huoshanense* residue was added to 300 mL double-distilled water (ddH_2_O) and extracted thrice at 100 °C for 4 h each. The mixture was centrifuged at 6000× *g* for 20 min to collect the supernatant, which was then concentrated and precipitated with 5 volumes of absolute ethanol and kept overnight at 4 °C. The precipitate was collected after centrifugation and dialyzed in ddH_2_O for 36 h, which was finally freeze-dried to produce crude DhPs. The contents of carbohydrates and protein were analyzed and calculated to be 82.9% and 8.7%, respectively.

### 3.3. Separation of DhPs and Proteins Using ILATPS

In order to assess the ability of [C_4_mim]Cl to extract proteins, different amounts of IL and inorganic salts (KOH, K_3_PO4, Na_2_CO_3_ and K_2_HPO_4_), and 1.0 mL BSA solution of different concentrations were mixed with 4.0 mL ddH_2_O in a 10 mL centrifuge tube. A blank control with all the constituents except for BSA was set for calibration. A vortex mixer was applied to ensure complete dissolution of all ingredients. The mixture was then incubated at a certain temperature for 1 h before subjected to centrifugation at 8000× *g* for 10 min to form two distinct phases. As a result, IL and proteins were concentrated in the upper layer comprising a small volume. On the other hand, the lower phase, occupying a large volume, was a salt-rich solution capable of dissolving carbohydrates. The two layers were separated using pipettes and the respective volumes recorded. The presence of remaining BSA in the lower phase was quantified to calculate the extraction efficiency of [C_4_mim]Cl.

**Figure 7:**
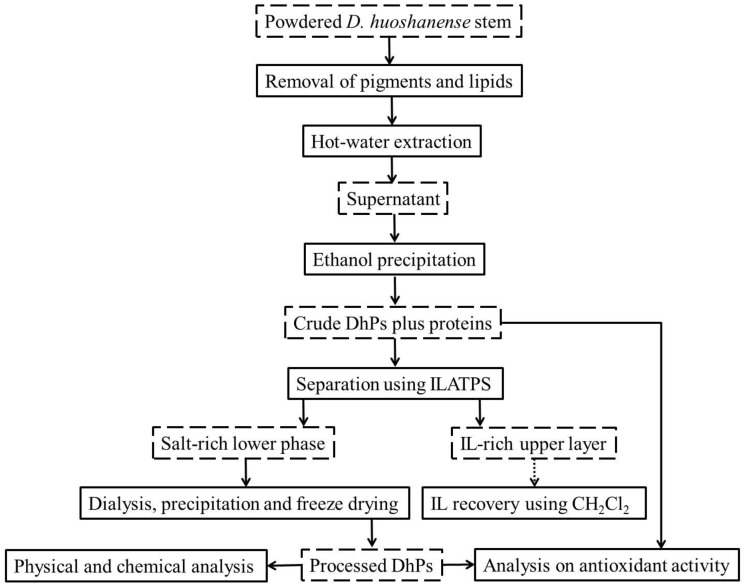
Flowchart for separation of *D. huoshanense* polysaccharides and proteins.

**Figure 8:**
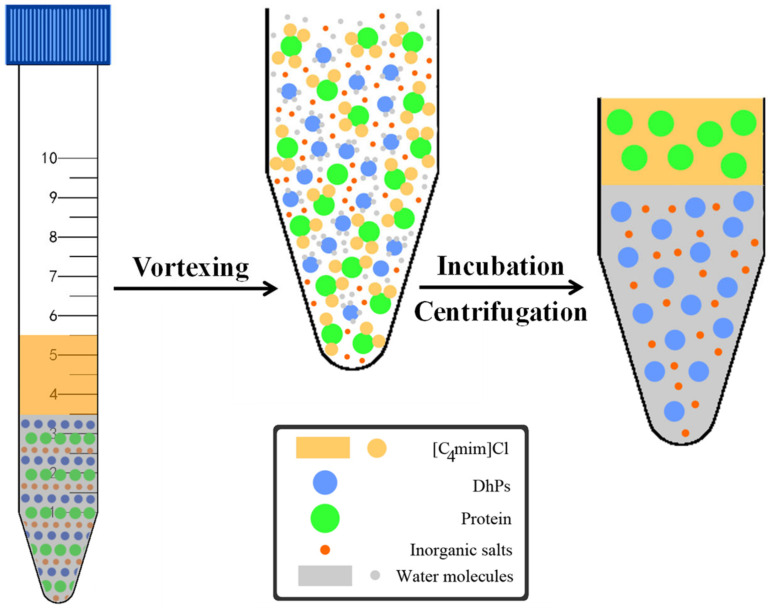
Extraction procedure and mechanisms of DhPs and protein separation in ILATPS.

With the best combination of extraction conditions in place, BSA was substituted with crude DhPs for further study. The lower phase of the solution was dialyzed (molecular weight cut off 8 kDa) in deionized water for 36 h before precipitated by absolute ethanol. The precipitate was freeze-dried to yield processed DhPs for subsequent physical and chemical analysis. Given the fact that polysaccharides are the main bioactive substance in *D. huoshanense*, analysis of the antioxidant activity was carried out for both crude and processed DhPs. Figure 7 as well as Figure 8 demonstrated the experimental pipeline of ILATPS and the mechanisms ensuring efficient separation of target compounds.

### 3.4. Assessment of DhPs and Protein Concentrations in ILATPS

Concentrations of both DhPs and proteins were determined by phenol-sulfuric acid and the Bradford approach, respectively [34,35]. The method to prepare the Coomassie Brilliant Blue G-250 solution was slightly modified. The dye (25 mg) was dissolved in 25 mL methanol to make a dark blue solution to which 42.5 mL phosphoric acid was added and, subsequently, 432.5 mL ddH_2_O was mixed with the previous solution. The extraction efficiency of DhPs was determined by calculating the total amount of carbohydrate (m_c-total_) in the crude DhPs solution, concentration of DhPs (C_c-low_) as well as volume (V_low_) in the lower phase using the following equation: [(C_c-low_ × V_low_)/m_c-total_] × 100%. Regarding protein extraction efficiency, the total amount of *D. huoshanense* protein (m_p-total_) in the crude DhPs solution, and the concentration of protein (C_p-low_) and volume (V_low_) in the lower phase were used for calculation adopting this equation: [1-(C_p-low_ × V_low_)/m_p-total_] × 100%.

### 3.5. Analysis of Crude and Processed DhPs Antioxidant Activity

An amount of 4.0 mg DPPH was weighed and dissolved in a 100 mL volumetric flask filled with a suitable amount of absolute ethanol to achieve a concentration of 0.004% DPPH. The solution was kept in the dark at 4 °C. Different concentrations of crude and processed DhPs (0, 0.1, 0.2, 0.4, 0.6 and 0.8 mg/mL) were prepared. For each reaction, DPPH solution (2.0 mL) was added to 2.0 mL DhPs samples and mixed thoroughly. Vc and ddH_2_O were used as the positive and blank control, respectively. The mixtures were kept in the dark for 30 min before their absorbance (Abs) was recorded at 517 nm. The scavenging capacity was calculated with the following equation: [(Abs of blank − Abs of sample)/Abs of blank] × 100%.

### 3.6. Determination of DhPs Homogeneity and Molecular Weight

The homogeneity and molecular weight of processed DhPs were analyzed using HPGPC conducted on a Waters 1515 GPC system equipped with a Waters 2410 refractive index detector (Waters, Milford, MA, USA) and a Shodex OHpak SB-804 HQ column (8 × 300 mm, 7μm). The following are the chromatographic conditions: a 45 min isocratic elution was applied with the flow rate of 0.6 mL/min; mobile phase was NaCl solution (0.05 M) with the column temperature set to 40 °C. NaCl solution (0.05 M) was used to dissolve processed DhPs whose final concentration was made to 5 mg/mL for analysis. Dextran polymer standards (Sigma-Aldrich, USA) with different molecular weights (1, 5, 12, 25, 50, 80 and 670 kDa) were used for molecular weight estimation. Data acquisition and determination were performed utilizing Empower Software (Waters, Milford, MA, USA).

### 3.7. DhPs Monosaccharide Composition Analysis

Processed DhPs (5 mg) were hydrolyzed with 1 mL of 2.0 M TFA at 120 °C for 2 h in a sealed tube and the solution was then dried with nitrogen. After rinsing with methanol 3 times, residue of hydrolyzed DhPs was dissolved in ddH_2_O for analysis. The samples and standards were analyzed using an ICS5000 ion chromatography system (Thermo Fisher, Waltham, MA, USA) equipped with a pulsed amperometric detector. The column was Dionex CarboPac PA20 (150 × 3.0 mm, 10 μm) and the column temperature was 30 °C. An A solvent containing 0.1 M NaOH in water and B solvent containing 0.1 M NaOH and 0.2 M NaAc in water were used to produce a gradient profile from 5-20-40-40-5-5 % B from 0.0-30.0-30.1-45.0-45.1-60.0 min, at a flow rate of 0.5 mL/min. After analysis, chromatographic peaks of respective monosaccharides were manually integrated and the concentrations were calculated using an external calibration curve fitted with linear regression.

### 3.8. FT-IR Spectra Analysis

Processed DhPs (2.0 mg) were ground thoroughly with 200 mg KBr (Aladdin, Shanghai, China), which was then pressed into a disk for analysis. A Nicolet 6700 Fourier-transform infrared (FT-IR) spectrometer (Thermo Fisher, USA) was applied to determine FT-IR spectra of the target compounds with the wavenumber ranging from 400 to 4000 cm^−1^.

### 3.9. [C_4_mim]Cl Recycling

To avoid environmental contamination, the IL-rich upper layer was extracted with CH_2_Cl_2_ to recover [C_4_mim]Cl. In this study, the IL-rich phase containing [C_4_mim]Cl was first concentrated using a water bath with the temperature set to 98 °C to remove the water, and then the residue was dissolved into a CH_2_Cl_2_ solution, which will be evaporated in a ventilator to recover the IL. CH_2_Cl_2_ causes denaturation and precipitation of protein, so the recycled IL can be reused for the separation of protein and polysaccharides.

### 3.10. Data Analysis

Statistical analysis was conducted using GraphPad Prism 7.00 software. Student’s *t*-test or one-way analysis of variance (ANOVA) was used to determine statistical significance with a *p* value less than or equal to 0.05 deemed as statistically significant.

## 4. Conclusions

Efficient separation of proteins and polysaccharides extracted from *D. huoshanense* using [C_4_mim]Cl-based partitioning system was achieved. By vortexing, water and the IL are mixed thoroughly. Hydrogen bonding interactions occur between water molecules and DhPs, and electrostatic or hydrophobic interactions let the IL stick together with proteins [25]. After incubation and centrifugation, proteins and carbohydrates are separated into the upper and lower phases respectively (Figure 8).

With the optimal conditions of ILATPS (1.75 g K_3_PO_4_, 1.25 g [C_4_mim]Cl, 10 mg crude DhPs and 5.0 mL ddH_2_O at pH 7.0 under 25 °C), extraction efficiency rates for DhPs and proteins were 93.4% and 90.2%, respectively (Figure 2D). The DhPs retrieved were further purified by dialysis, ethanol precipitation, freeze-drying and subjected to physical and chemical analysis. The processed DhPs contained Man, Glc, Gal, Ara and Gal-UA with a molar ratio of 185:71:1.5:1:1 and the Mw was 2.14 × 10^5^ Da. This approach is fast, simple and environment friendly. It provides a new insight into purifying functional polysaccharides of plant origin.

## Data Availability

Not applicable.

## Sample Availability

Samples of the plant material are available from the authors.
